# 1367. COVID-19 Patients Expressed Distinct Clinical Trait Signatures at Index Hospitalizations Across the Four *SARS-CoV-2* Pandemic Waves in Florida

**DOI:** 10.1093/ofid/ofad500.1204

**Published:** 2023-11-27

**Authors:** Ian Motie, John Sia, Joseph Seaman, Karen Hamad, Wilhelmine Wiese-Rometsch, Robert A Smith, Manuel E Gordillo

**Affiliations:** Florida State University Internal Medicine Residency at Sarasota Memorial Hospital, Sarasota, Florida; Florida State University Internal Medicine Residency at Sarasota Memorial Hospital, Sarasota, Florida; Sarasota Memorial Hospital, Sarasota, Florida; FSU SMH Internal Medicine Residency Program, Sarasota, Florida; Sarasota Memorial Health Care System, Sarasota, Florida; Florida State University College of Medicine, Sarasota, Florida; Sarasota Memorial Hospital, Sarasota, Florida

## Abstract

**Background:**

Adverse clinical outcomes have been associated with COVID-19 mortality, but predictive stability across *SARS-CoV-2* pandemic variants has not been reported. Machine learning identified clinical traits and their relative importance independently associated with mortality at index hospitalization. The purpose was to compare variance explained (VE%) by each trait during pandemic Wave 1: March 10, 2020 – June 18, 2021; Wave 2: June 19, 2021 – December 18, 2021; Wave 3: December 19, 2021 – March 30, 2022; and Wave 4: March 31, 2022 – April 14, 2023, at an 818-bed academic safety-net hospital.

**Figure 1:** SARS-CoV-2 Tests from March 20, 2020 to April 14, 2023

**Figure:**
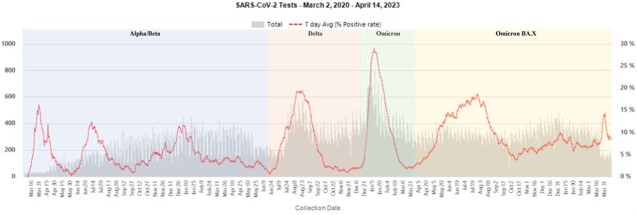

Collected positive SARS-CoV-2 test results with color-shaded areas representing "waves" based on surges in positive tests and 7-day average positive rate. Discrete waves are titled based on the current predominant variant.

**Methods:**

Demographics, laboratory results, ICD-10-CM-based comorbidity, COVID-19 directed treatment and administrative data were extracted under IRB exemption from electronic medical records. Generalized regression with adaptive LASSO identified traits associated with mortality controlling for COVID-19 directed treatment in at least one of the waves. Univariate logistic regression for each trait created a within-variant receiver operating characteristic curve with optimal cut-point (Youden Index) associated with mortality. Boosted Tree computed within-variant proportion (VE%) contributed by clinical traits in the model’s representation (R^2^) of mortality risk. Continuous data summarized with median [IQR] were compared using Kruskal-Wallis. Discrete data summarized as proportions were compared with chi-square.

**Results:**

6490 patients were distributed across Wave 1 (2249), Wave 2 (1196), Wave 3 (953) and Wave 4 (2092) with respective mortality of 3%, 2%, 1%, and 1% (p< .0001). The four waves were titled Alpha/Beta, Delta, Omicron, and Omicron BA.X based on predominant strains during dates of admissions. Table 1 displays patient demographics in each of the four waves. Tables 2-5 display VE% for 29 clinical traits in each respective wave.

**Table 1:**
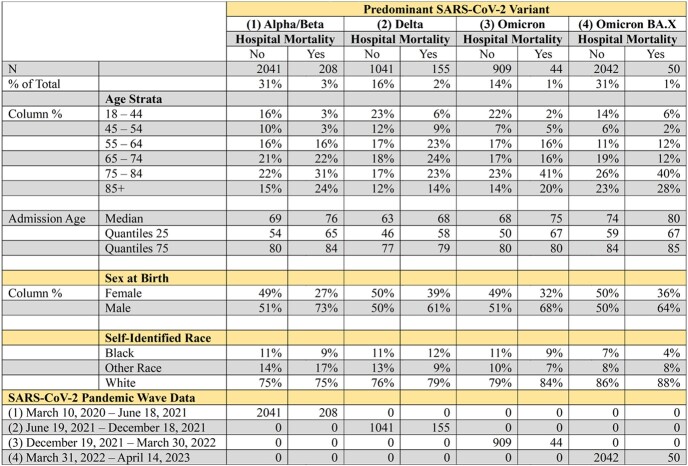
COVID-19 Patient Demographics. Demographics for patients admitted with COVID-19 infection. Demographics are compared based on Pandemic Wave.

**Table 2:**
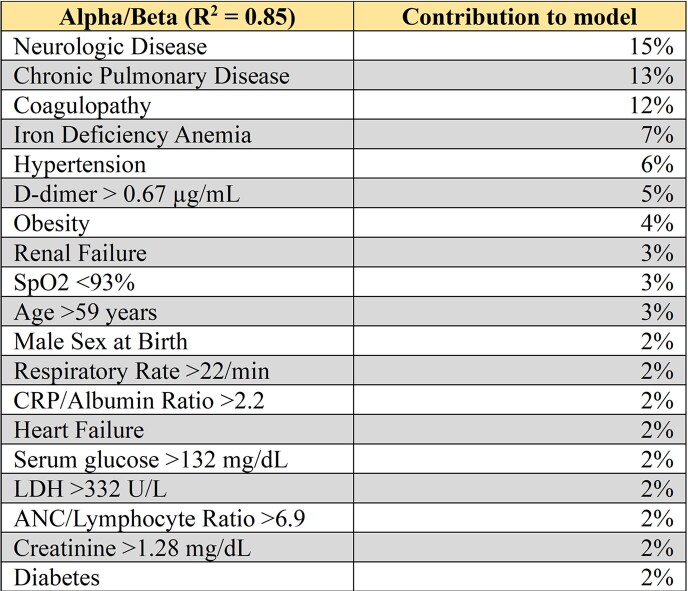
Trait signature during Alpha/Beta Wave. Contribution of twenty-nine different clinical traits to Alpha/Beta Wave model.

**Table 3:**
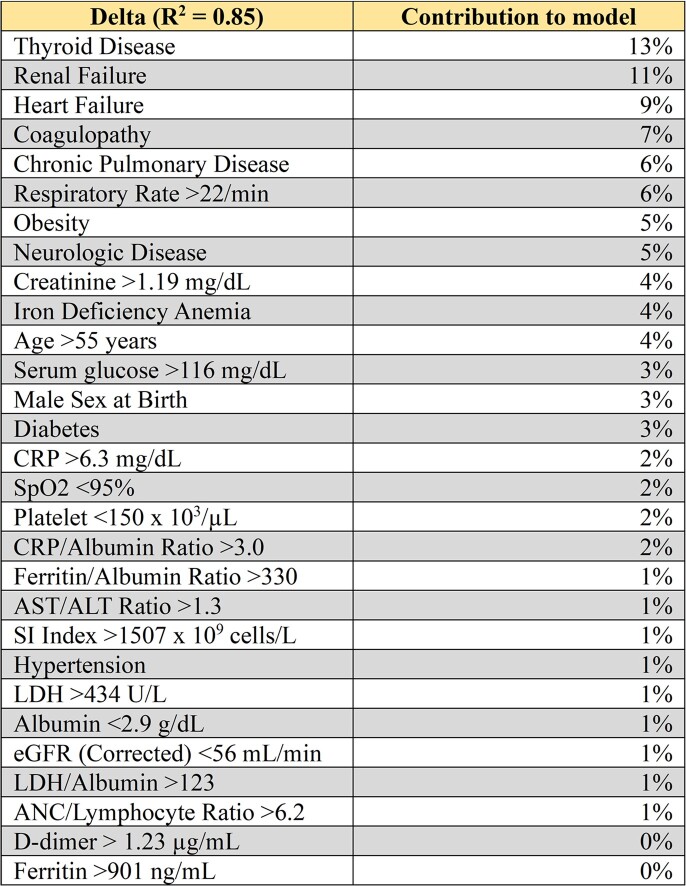
Trait signature during Delta Wave. Contribution of twenty-nine different clinical traits to Delta Wave model.

**Conclusion:**

The developed longitudinal training models suggest that COVID-19 presentation risk tools may not generalize across pandemic surges. Limitations includes monocenter study with many patients self-reporting vaccination status. Study strength includes demonstrating utility of longitudinal machine modeling as a tool for learning healthcare system science. Future research may further examine co-morbid conditions and presentation severity.

**Table 4:**
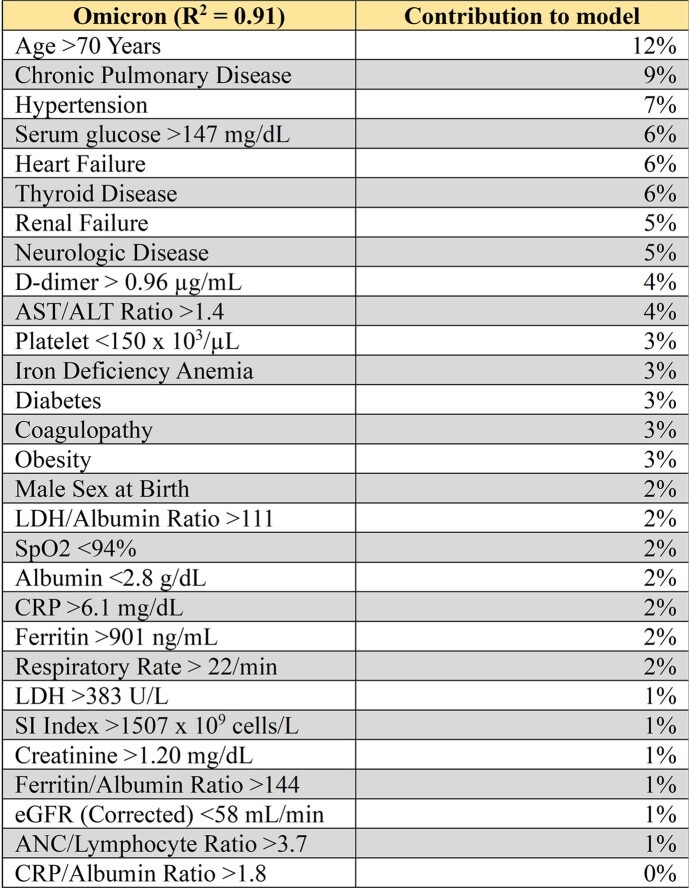
Trait signature during Omicron Wave. Contribution of twenty-nine different clinical traits to Omicron Wave model.

**Table 5:**
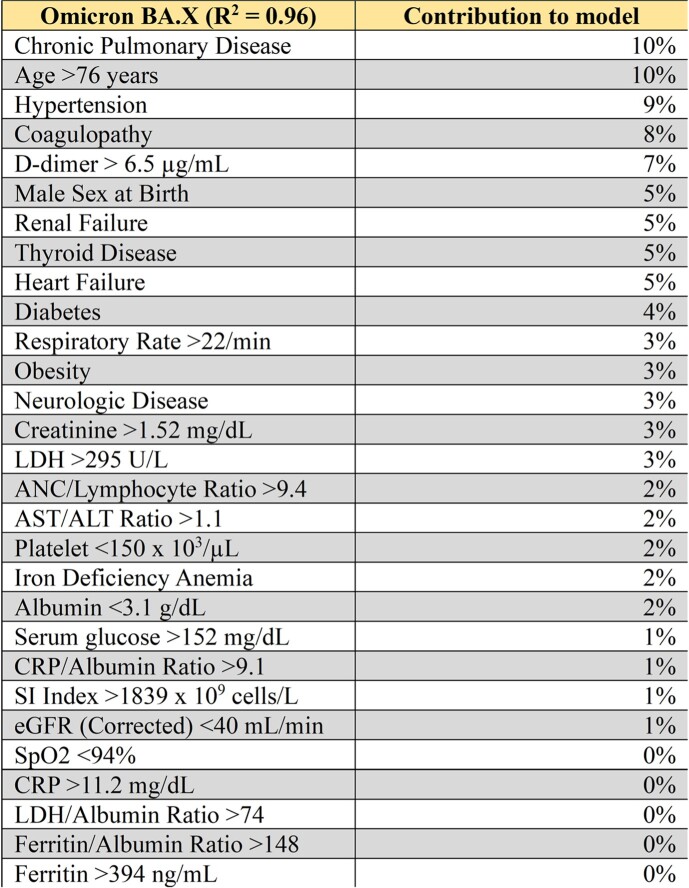
Trait signature during Omicron BA.X Wave. Contribution of twenty-nine different clinical traits to Omicron BA.X Wave model.

**Disclosures:**

**Manuel E. Gordillo, M.D.**, Regeneron: Was involved in the Regen Cov trial as a PI one one of the sites

